# Developmental studies provide new insights into the evolution of sense organs in Sabellariidae (Annelida)

**DOI:** 10.1186/s12862-018-1263-5

**Published:** 2018-10-04

**Authors:** Conrad Helm, Michael J Bok, Pat Hutchings, Elena Kupriyanova, María Capa

**Affiliations:** 1 Animal Biodiversity and Evolution, University of Goettingen, Untere Karspüle 2, 37073 Goettingen, Germany; 2 School of Biological Sciences, University of Bristol, Life Sciences Building, 24 Tyndall Avenue, Bristol, BS8 1TQ UK; 3 Australian Museum Research Institute, Australian Museum, 1 William Street, Sydney, NSW 2010 Australia; 4 Biological Sciences, Department of Biological Sciences, Macquarie University, North Ryde, NSW 2109 Australia; 5 Biology Department, University of the Balearic Islands, Department of Biology, Ctra. Valldemossa, Palma de Mallorca, Balearic Islands Spain; 6 NTNU University Museum, Norwegian University of Sciences and Technology, NO-7491 Trondheim, Norway

**Keywords:** Sensory organs, Polychaetes, Sedentaria, Settlement, Larva, Median organ

## Abstract

**Background:**

Sabellarids, also known as honeycomb or sandcastle worms, when building their tubes, produce chemical signals (free fatty acids) that are responsible for larval settlement and the formation of three-dimensional aggregations. The larval palps and the dorsal hump (becoming the median organ in adults) are presumed to participate in such a substrate selection during settlement. Notably, the sabellariid median organ is an apparently unique organ among annelids that has been attributed with a sensory function and perhaps with some affinities to the nuchal organs of other polychaetes. Nevertheless, detailed investigations of this prominent character complex including ultrastructural examinations are lacking so far.

**Results:**

Our comprehensive investigations provide data about the anterior sensory organs in Sabellariidae and inform about their transformation during pelagic larval development. We used a comparative approach including immunostaining with subsequent confocal laser scanning microscopy (clsm), histological sections as well as electron microscopy in a range of larval and adult stages of two sabellariid species. We find that the neuronal innervation as well as the ultrastructure of the sabellariid ciliary structures along the median organ are highly comparable with that of nuchal organs known from other polychaetes. Furthermore, the myoinhibitory protein (MIP) – a protein known to be also involved into chemo-sensation - was detected in the region of the larval median organ. Moreover, we reveal the presence of an unusual type of photoreceptor as part of the median organ in *Idanthyrsus australiensis* with a corrugated sensory membrane ultrastructure unlike those observed in the segmental ocelli of other polychaetes.

**Conclusions:**

We are describing for the first time the nuchal organ-like structures in different developmental stages of two species of Sabellariidae. The external morphology, neuronal innervation, developmental fate and ultrastructure of the newly-discovered median organ-based ciliary pits are comparable with the characteristics known for annelid nuchal organs and therefore indicate a homology of both sensory complexes. The presence of myoinhibitory peptide (MIP) in the respective region supports such a hypothesis and exhibits the possibility of an involvement of the entire sabellariid median organ complex, and in particular the prominent ciliated pits, in chemo-sensation.

**Electronic supplementary material:**

The online version of this article (10.1186/s12862-018-1263-5) contains supplementary material, which is available to authorized users.

## Background

Sabellariidae Johnston, 1865, also known as honeycomb or sandcastle worms, is a specialized group of marine segmented worms (Annelida) that live in tubes of cemented sand grains or biogenic particles. The anterior end of the worms is modified into an operculum with rows of golden paleae that can seal the entrance of the tube when the animal withdraws into it for protection or to avoid desiccation. Sabellariids are free-spawners releasing their gametes into the water column where the eggs become fertilized and have – like many other benthic taxa [1] - a long lasting planktotrophic larva for which the neuronal development is well investigated [2–5]. Some species are gregarious and build their tubes attached to other conspecifics, which results in three-dimensional structures that can extend over several kilometres in intertidal environments [5, 6]. Sabellariids have been the object of several studies aiming to decipher the molecular cues that induced gregarious settlement, and the sensory organs potentially involved in this process. Chemical signals are known to be involved in settlement behaviour [7–9]. For sabellariids, molecules present in the cement secreted by benthic juveniles and adults when building their tubes (free fatty acids), are known to be responsible for larval settlement [7, 10–15]. In this respect, the larval palps and the dorsal hump (becoming the median organ in the adults) are presumed to participate in substrate selection during settlement [16–18].

Nuchal organs are epidermal sensory structures, and one of the characteristic features of annelids, despite being reduced or lacking in some groups (e.g. Clitellates, members of Siboglinidae or basally branching taxa such as Magelonidae) [19]. Nuchal organs show a broad morphological variability among annelids. They are generally present as a pair of densely ciliated pits or areas at the posterior part of the prostomium [20], but in members of Amphinomidae the cililated pits run along the sides of a bulbous median structure called a caruncle, in some Spionidae and Syllidae the ciliary bands are found mainly along prostomial lobes, in some Phyllodocidae in a retractile tongue-like structure, and they form a pair of pouches arising from the dorsal epithelium of the mouth cavity in members of Fabriciidae and Sabellidae [20–24].

**Fig. 1:**
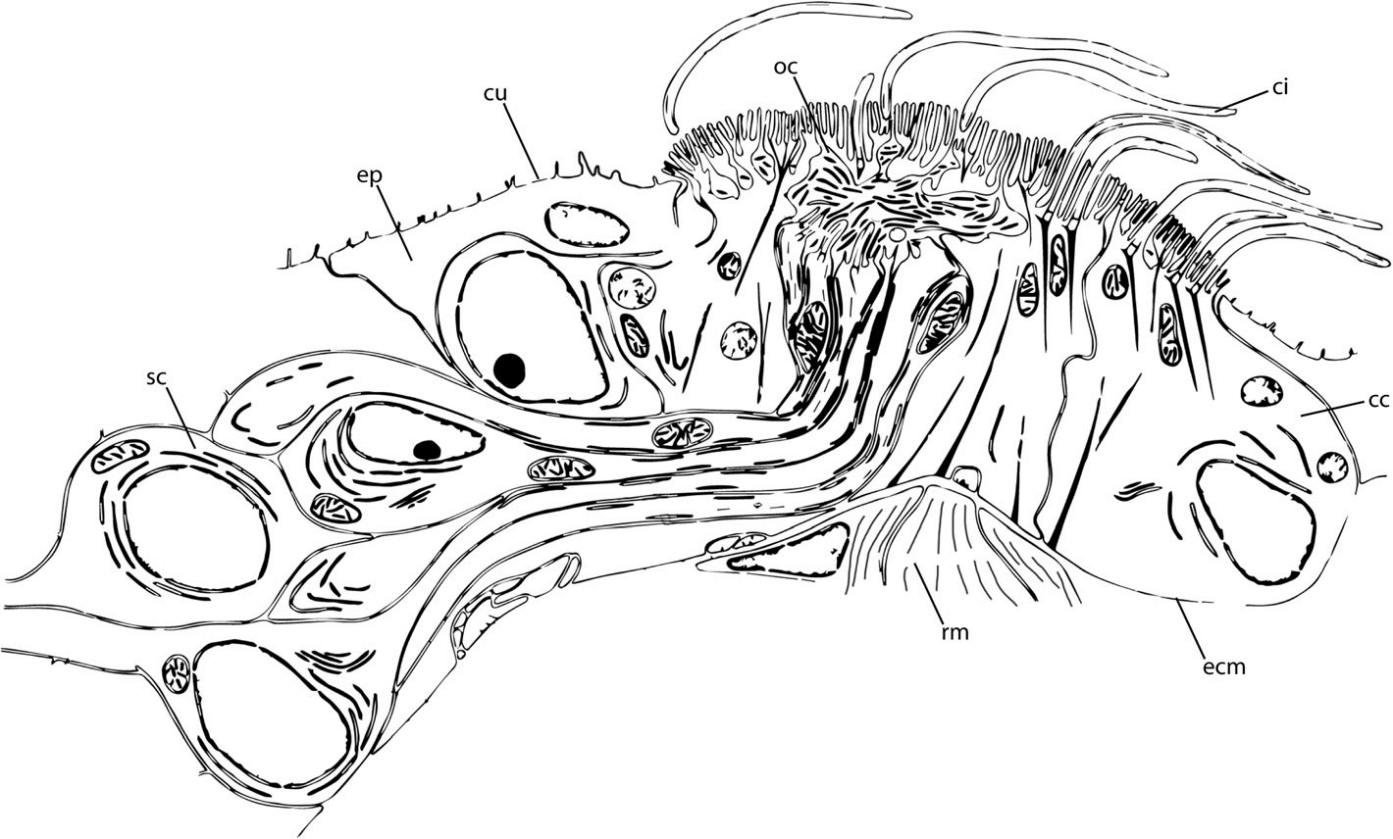
Schematic overview showing a nuchal organ of *Nerillidium troglochaetoides* (Nerillidae) illustrating the general organiszation of an annelid nuchal organ. The organ consists of ciliated supporting cells (sc) with microvilli and cilia (ci), an olfactory chamber (oc), and prominent sensory cells (sc) running towards the olfactory chamber (oc). The entire structure is embedded in the epidermis (ep) and can be retracted via a distinct retractor muscle (rm). cc, ciliated supporting cell; ci, cilium; cu, cuticle; ecm, extracellular matrix; ep, epidermis; oc, olfactory chamber; rm., retractor muscle; sc, sensory cells. The image was redrawn and modified from Purschke 1997 [20]

Nuchal organs consist of at least ciliated supporting cells and bipolar primary sensory cells. Sensory processes terminate in a subcuticular olfactory chamber overlaid by a specialised protective cuticular cover, and paired nerves innervate the organs directly from the posterior part of the brain [20, 24, 25]. A general overview of the annelid nuchal organ organization is given in Fig. 1. The nuchal organs, due to their ultrastructure, are considered to be chemoreceptors, but their specific function in many annelids where they have been described is yet to be assessed with physiological evidences [20, 22, 23, 26, 27]. Nevertheless, latest investigations in *Platynereis dumerilii* highly support such a chemosensory function [28]. In Sabellariidae, the ultrastructure of the nuchal organs has not been studied in detail, but its presence was assumed at the base of the palps, due to the innervation of a densely ciliated area by a nerve equivalent to the nuchal nerve associated with the nuchal organs in spionids and flabelligerids [29]. However, detailed studies of the palps in *Phragmatopoma californica* (Fewkes, 1889) did not find evidence of any of the elements typical of the nuchal organs [16].

Nevertheless, the median organ, a characteristic and apparently unique organ for Sabellariidae, of prostomial origin, digitiform in shape and located in the junction of the anterior opercular lobes, has also been attributed with a sensory function [10, 18, 30–32]. In fact, the larval dorsal hump, the incipient form of the adult median organ, has been suggested to play a major role in chemoreception for settlement [16–18]. Some sabellariid species also bear a band of presumed eyespots at both sides of the median organ [8, 10, 32, 33] but their ultrastructure and composition have not yet been studied so far.

In order to elucidate further details of morphological structures and the putative role of the sabellariid dorsal hump/median organ, we examined various developmental stages of the sabellariid species *Sabellaria alveolata* (Linnaeus, 1767) and *Idanthyrsus australiensis* (Haswell, 1883) using a comparative approach with diverse microscopic techniques including immunostaining with subsequent confocal laser scanning microscopy (clsm), as well as histological sections and electron microscopy. Sabellariid species with dissimilar median organs (morphology and size) were selected to cover the variability of forms in the group. *Sabellaria alveolata* (Linneaus, 1767) and *Idanthyrsus australiensis* (Haswell, 1883) were selected as sabellariid representatives with a small and well-developed median organ in adults, respectively [18]. Furthermore, both species build small aggregates to large reefs (especially *Sabellaria*) [8] although *I. australiensis* can occur as solitary individuals.

Our comprehensive investigations provide further necessary data on the anterior sensory organs in Sabellariidae and their transformation during a pelagic larval development.

## Methods

### Larval culture and fixation

Adult specimens of *S. alveolata* were collected at Saint-Efflam (Brittany, France) in June 2015, transferred to Bergen (Norway) and reared in a lab-based seawater cycle at 14–17 °C.

The adult *S. alveolata* for electron microscopy were collected near Bude (UK) in March 2017. Adult specimens of *I. australiensis* were collected at Long Reef, Sydney (New South Wales, Australia) in January 2017 and subsequently reared in filtered seawater at 20 °C. After artificial fertilization in filtered seawater (FSW), developmental stages were reared at 18 °C (for *Sabellaria*) or 20 °C (for *Idanthyrsus*) in glass bowls containing FSW. The cultures were set under strict diurnal rhythm (14 h (h) light: 10 h dark) and fed with a mix of unicellular algae (*Tisochrysis lutea*, *Isochrysis* spp.*, Chaetoceros* spp.). Water was changed regularly. Developmental stages were defined by the number of “days post fertilization” (dpf) required for the majority of larvae to reach a specific developmental point.

### Fixation of different stages

Larval stages of both species were anaesthetized using 7% MgCl_2_ in FSW. Subsequently stages were fixed in 4% paraformaldehyde (PFA) in 1× phosphate buffered saline (PBS) containing Tween (1× PBS: 0.05 M PB/0.3 M NaCl/0.1% Tween20) for 1 h at room temperature (RT). Afterwards, specimens were rinsed in 1× PBS several times and stored in 1× PBS containing 0.05% NaN_3_ at 4 °C until usage.

For electron microscopy, larvae were fixed in 2.5% glutaraldehyde in sodium cacodylate buffer (0.1 M Cacodylate, pH 7.4, 0.24 M NaCl) for 1 h at RT. Adult anterior ends of *S. alveolata* were fixed in 2% paraformaldehyde (PFA), 2.5% glutaraldehyde, 2% sucrose in 0.1 mol − 1 Sörensen’s phosphate buffer, pH 7.2, for 1 h at room temperature and stored in 0.1 mol − 1 Sörensen’s phosphate buffer.

### Scanning electron microscopy (SEM)

For larval SEM analyses, 10 specimens of each investigated stage and species were used. The samples were washed 2 × 5 min in sodium cacodylate buffer without addition of NaN_3_. Afterwards, the specimens were postfixed in osmium tetroxide (1% O_S_O_4_ in sodium cacodylate buffer) for 45 min at RT and rinsed with distilled water for 3 × 10 min. Subsequently, the samples were dehydrated using an increasing EtOH series (30–40%-50–60%-70–80%-90–95%-3 × 100%, 5–10 min each), critical-point dried and coated with gold/palladium. Finally, the samples were investigated with a Supra 55VP scanning electron microscope (Zeiss, Germany). Alternatively, the dehydrated specimens (100% EtOH) were placed in an ascending series (25–50%-75-2 × 100%, 15 min each) of hexamethyldisiloxane (HMDS) and then air dried. The prepared samples were mounted on holders and sputter-coated with gold. The micromorphology and topography were determined using a Philips FEIINSPECT (Hillsboro, Oregon, USA) and a JEOL 6480LA SEM. The samples were observed with the Back Scattering Electron Detector (BSED). The final panels were designed using Adobe (San Jose, CA, USA) Photoshop CC and Illustrator CC.

### Transmission electron microscopy (TEM)

Pre-fixed anterior ends of adult specimens of both species were washed in phosphate buffer (0.1 mol l^− 1^) followed by 1% osmium tetroxide in phosphate buffer for 1 h at 4 °C. Samples were washed in phosphate buffer, dehydrated by an ethanol series, infiltrated with acetone and Epon plastic (Electron Microscopy Sciences, Hatfield, PA), and embedded in Epon. Ultra-thin TEM sections (50–70 nm) were produced using a Leica (Wetzlar, Germany), ultracut with a diamond knife and stained with 2% uranyl acetate and lead citrate. Sections were viewed on a JEM-1230 TEM (JEOL, Glen Ellyn, IL). The final panels were designed using Adobe (San Jose, CA, USA) Photoshop CC and Illustrator CC.

### Immunohistochemistry and confocal laser scanning microscopy (clsm)

Antibody stainings using standard markers as well as the antibody against the anti-myoinhibitory peptide (MIP) were revealed in whole animal preparations. The antibody directed against anti-myoinhibitory peptide (MIP) was used due to the involvement of the peptide into annelid chemo-sensation and larval settlement [34]. Although the specificities of the used antibodies have all been established in numerous invertebrates (for references see Discussion), we cannot exclude that a given antiserum may bind to a related antigen in the investigated specimens. Therefore, we refer to observed profiles as exhibiting (antigen-) like immunoreactivity (-LIR). Negative controls were obtained by omitting the primary antibody in order to check for antibody specificity and yielded no fluorescence signal.

At least 20–30 specimens of each species and all developmental stages were investigated for immunohistochemistry. Individuals were rinsed 2 × 5 min in PTW (PBS with 0.1% Tween 20) at room temperature (RT) and subsequently transferred into 10 μg proteinase K/ml PTW for 1–4 min depending on the developmental stage and size. After two short rinses in glycine (2 mg glycine/ml PTW), and 3 × 5 min washes in PTW, specimens were fixed a second time using 4% PFA in PBS containing 0.1% Tween for 20 min at RT, rinsed 2 × 5 min in PTW, 2 × 5 min in THT (0.1 M Tris-HCl pH 8.5, 0.1% Tween-20) and blocked for 1–2 h in 5% sheep serum in THT. The primary antibodies, rabbit anti-myoinhibitory peptide (MIP) ([34], dilution 1:150), polyclonal rabbit anti-5-HT (INCSTAR, Stillwater, USA, dilution 1:500) and monoclonal mouse anti-acetylated α-tubulin (Sigma-Aldrich, St. Louis, USA, dilution 1:250), were applied for 48–72 h in THT containing 5% sheep serum at 4 °C. Afterwards, specimens were rinsed twice in 1 M NaCl in THT, then washed 5 × 30 min in THT and incubated subsequently with secondary fluorochrome conjugated antibodies (goat anti-rabbit Alexa Fluor 488, Invitrogen, USA, dilution 1:500; goat anti-mouse Alexa Fluor 633, ANASPEC, Fremont, USA, dilution 1:500) in THT containing 5% sheep serum for 48 h at 4 °C. Subsequently, samples were washed 6 × 30 min in THT, stained with DAPI for 15–30 min (5 mg/ml stock solution, working solution: 2 μl in 1 ml THT – final concentration 10 μg/ml) and washed 2 × 5 min in THT. Specimens were then dehydrated using an ascending ethanol series, transferred into Murray’s Clear solution (2 parts benzyl benzoate and 1 part benzyl alcohol) and subsequently mounted between two cover slips using DPX slide mounting medium (Sigma-Aldrich, St. Louis, USA). Specimens were analysed with the confocal laser-scanning microscope Leica TCS SP5 (Leica Microsystems, Wetzlar, Germany). Confocal image stacks were processed with Leica AS AF v 2.3.5 (Leica Microsystems) and Imaris 8.3 (Bitplane AG, Zurich, Switzerland). The final panels were designed using Adobe (San Jose, CA, USA) Photoshop CC and Illustrator CC.

### Histology and semi-thin sections

Specimens fixed in 4% PFA were embedded in paraffin (LeicaEG1160), sectioned (4 μm, with a Leica RM2255 microtome) and dried at 60 °C. Haematoxylin Erythrosine Saffron (HES) staining was performed in the automatic slide stainer SakuraTissue-Tek©Prisma™. The slides were dried further in the instruments heat chamber, then de-paraffinized through several baths in Tissue Clear (Sakura, AlphenaandenRijn, Netherlands) and rehydrated through a descending ethanol series. Staining with haematoxylin was followed by bluing in water. The slides were stained in erythrosine and rinsed in water for removal of excess dye. Subsequently, samples were dehydrated through an ascending series of ethanol and stained in saffron (Chemi-Teknic as, Chroma), rinsed in several baths of absolute ethanol and cleared in Tissue Clear before cover slipping in the Sakura Tissue-Tek©Glas™ automatic coverslipper. For toluidine-blue staining, sections were transferred to glass slides, stained with toluidine blue (1% toluidine blue, 1% sodium tetraborate and 20% sucrose) and mounted with Depex. The sections were dried overnight. Photographs were taken with a Leica DFC 420 camera attached to a DM6000B compound microscope (Leica Microsystems, Wetzlar, Germany). The final panels were designed using Adobe (San Jose, CA, USA) Photoshop CC and Illustrator CC.

## Results

In the following descriptions we refer to the naming of morphological structures used previously [18] and the neuroanatomical vocabulary according to Richter et al. [35]. Differing definitions are stated appropriately.

### Development and neuronal innervation of the median organ in Sabellariidae

**Fig. 2:**
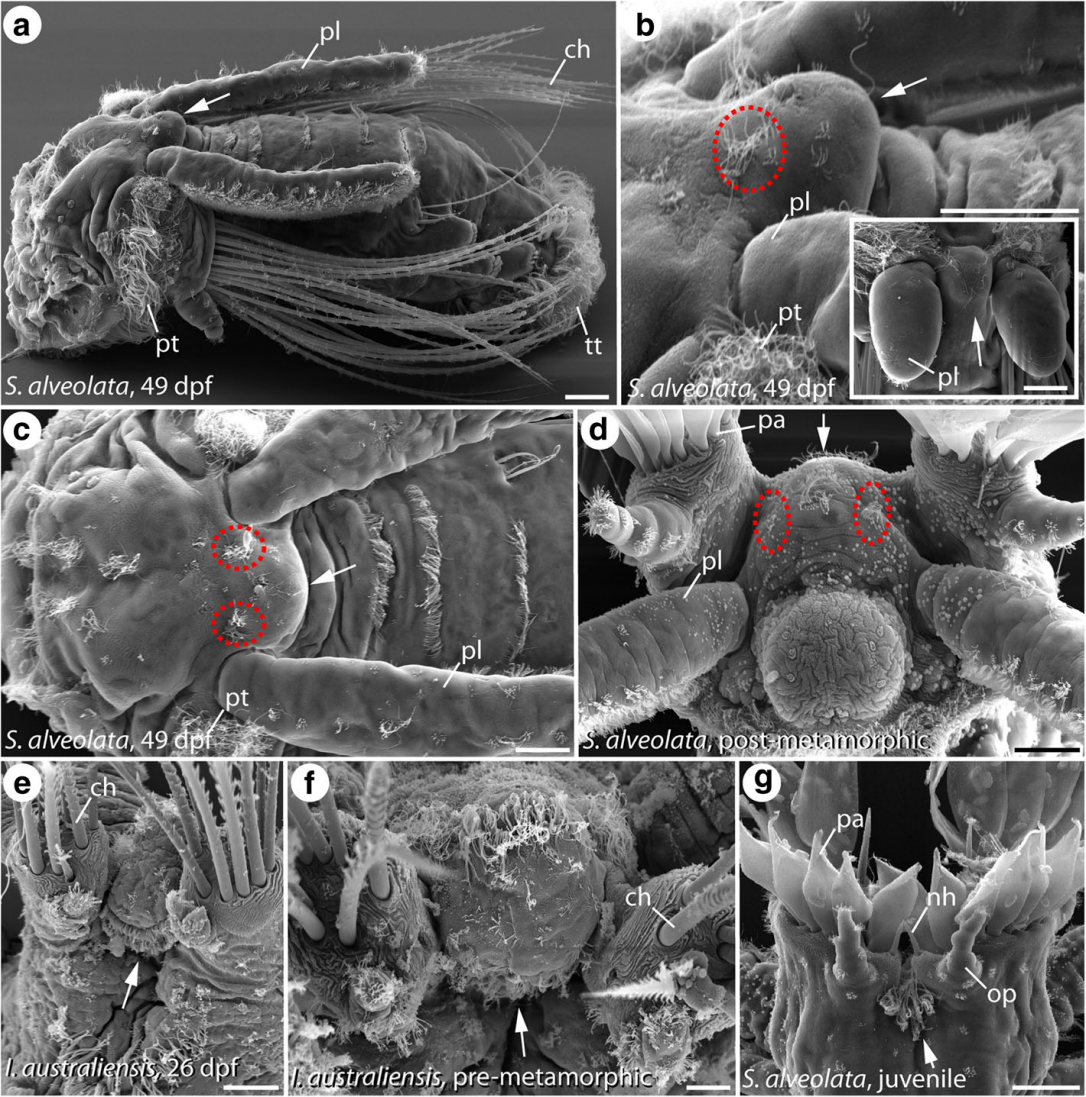
Scanning electron microscopic images of developmental stages of *Sabellaria alveolata* (**a**-**d**, **g**) and *Idanthyrsus australiensis* (**e**, **f**). Age of the larvae is given in days post fertilization (dpf) and the species identity is specified in the respective figure. In (**a**-**c**) anterior is left, in (**e**-**f**) anterior is up. **d** is a frontal view with the dorsal side up. **a** and **b** show a view from lateral. The inset in (**b**) shows a dorsal view with the anterior side up. **c** and (**e**-**g**) are dorsal views. The position of the larval median organ is marked by the white arrow and the position of the prominent ciliated tufts on the median organ is indicated by red dashed circles. **a** In pre-metamorphic larvae the prototroch (pt), telotroch (tt), the chaetae (ch) and the palps (pl) are well developed. The larval median organ is situated within the dorsal gap of the prototroch (pt), between the bases of the palps (pl). **b** A closer view reveals prominent ciliated tufts (red dashed ovals) and few additional cilia covering the surface of the larval median organ. The inset shows the same region in earlier larvae (~ 25 dpf) with palp (pl) buds already developing. Note the onset of the ciliation along the median organ. **c** A dorsal view reveals the position of the ciliated tufts at the base of the median organ. **d** In post-metamorphic specimens, the entire complex of the median organ is shifted from a dorsal towards a frontal position. The entire structure becomes flattened and incorporated in the formation of the juvenile anterior end. Nevertheless, the ciliated tufts (red dashed circles) are still detectable. **e** 26 dpf larvae of *I. australiensis* exhibit a median organ with a densely ciliated base. **f** The median organ is still well-developed even in larvae prior to metamorphosis. **g** In juveniles of *S. alveolata* the larval median organ is reduced in size, but still present as part of the anterior juvenile end. ch, chaetae; nh, nuchal hooks; op, opercular papillae; pa, palae; pl, palps; pt., prototroch; tt, telotroch. Scale bars: 20 μm (**a**-**e**), 10 μm (**f**) and 100 μm (**g**)

Late pre-metamorphic sabellariid larvae – starting from ~ 35 dpf in *Sabellaria alveolata* and ~ 14 dpf in *Idanthyrsus australiensis* - exhibit a prominent prototroch with underlying metatroch as well as primordial palps first recognizable as minute buds located close to the dorsal gap of the prototroch (Fig. 2b (inset)). Within this dorsal gap of the ciliated band, a protuberance develops and forms a prominent bulge in later larvae of *Sabellaria* and *Idanthyrsus*: the dorsal hump/median organ (Fig. 2a-c, e, f). In both investigated species, the larval median organ is best developed prior to metamorphosis (Fig. 2c, f). In this stage, a well-recognizable and locally dense ciliation on the median organ is observable (Fig. 2b, c, f). In particular, in pre-metamorphic specimens of *S. alveolata* at least two ciliated tufts are present on the dorsal surface of the median organ (Fig. 2b-d).

After metamorphosis, the median organ bulge stays prominent in *Idanthyrsus*, but becomes more flattened in *Sabellaria* (Fig. 2d). Furthermore, the position of the median organ in *Sabellaria* moves anteriorly and changes the position from dorsal towards frontal, and the entire median organ incorporates into the adult anterior end (Fig. 2d, g). Nevertheless, the distinct ciliated tufts visible in earlier larvae are still present in post-metamorphic stages (Fig. 2d).

**Fig. 3:**
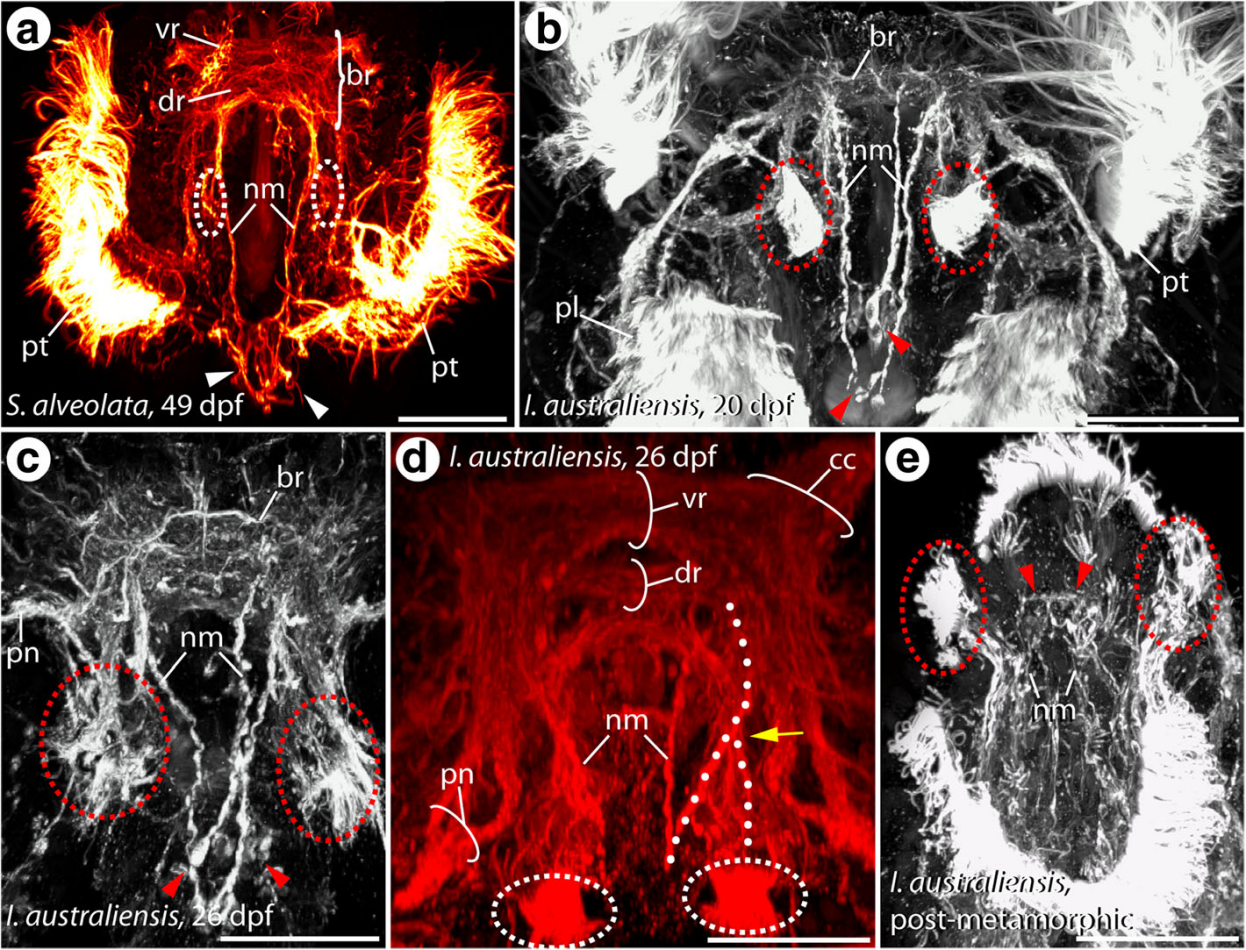
Development of the neuronal innervation (α-tub-LIR) of the larval median organ in *S. alveolata* (**a**) and *I. australiensis* (**b**-**d**). Confocal maximum projections. Anterior is up in all images. All views are dorsal, except of (**d**) which shows a view from apical. The position of the distinct ciliated tufts of the median organ is indicated by dashed circles and prominent perikarya at the tip of the median organ are marked with arrowheads. Age of the larvae is given in days post fertilization (dpf) and the species identity is specified in the respective figure. **a** The prominent prototroch (pt) as well as the innervating prototroch nerve (pn) are present in pre-metamorphic larvae of *S. alveolata*. The larval brain (br) consists of a ventral (vr) and dorsal root (dr) of the circumesophageal connective. Prominent neurite bundles innervating the median organ (nm) are detectable in the dorsal part of the brain. The latter bundles branch of from the dorsal root (dr) and run towards the median organ tip, where they terminate in distinct immunoreactive perikarya (arrowheads). At the base of the median organ distinct ciliated tufts (dashed circles) are present. **b** In pre-metamorphic stages of *I. australiensis* a comparable situation can be found. In these larvae the neurite innervating the ciliated tufts (dashed circles) branches of from the neurite bundles innervating the median organ (nm). **c** 26 dpf the bifurcation between (nm) and the neurite running towards the ciliated tuft is obvious. **d** The neurite bundles innervating the median organ branch off in a region closely related to the dorsal root of the circumesophageal connective (dr) and run towards the median organ tip. A prominent bifurcation (arrowhead) connects the ciliated tufts (dashed circles) with the later bundles. **e** In post-metamorphic stages the prominent neurite bundles innervating the median organ (nm), the apical immunoreactive perikaya as well as the ciliated tufts (dashed circles) are still present. Note that the entire median organ complex has changed its position during metamorphosis – from a dorsal towards a frontal orientation. br, brain; cc, circumesophageal connective; dr, dorsal root of the circumesophageal connective; nm, neurite bundle innervating the median organ; pl, palps; pn, palp nerve; pt., prototroch; vr, ventral root of the circumesophageal connective. Scale bars = 80 μm (**a**), 60 μm (**b**, **c**) and 30 μm (**d**, **e**)


When it comes to the development of the neuronal innervation of the median organ in both species, a comparable situation is observable. Thus, immunohistochemical staining against α-tubulin reveals the presence of α-tubulin (α-tub) -like immunoreactivity (-LIR) in the region of interest. In late pre-metamorphic larvae of both species, two distinct neurite bundles formed by at least two sub-bundles each and exhibiting α-tub-LIR branch - closely related to the dorsal root of the circumesophageal connective - from the dorsolateral larval brain (Fig. 3a, b and Additional file 1: Figure S1). Subsequently, these neurite bundles run towards the tip of the larval median organ, where both bundles terminate in a region bearing numerous perikarya showing α-tub-LIR (Fig. 3a, b (arrow heads) and Additional file 1: Figure S1). Notably, a prominent bifurcation of the neurite bundles coming from the dorsolateral part of the brain can be examined (Fig. 3d). Whereas one main branch of this bifurcation runs towards the median organ tip (as described above), the second branch runs more laterally and terminates in a densely ciliated tuft (Fig. 3a-e (dashed ovals)). In all investigated median organ-bearing pre-metamorphic stages of both species, two of such prominent and densely ciliated tufts with the described neuronal innervation are present (Fig. 3a-d and Additional file 2: Figure S2). Even after metamorphosis, the distinct tufts as well as the perikarya of the median organ tip and the prominent innervating neurite bundles are detectable – now in an erected and frontally-directed position (Fig. 3e and Additional file 3: Figure S3).

**Additional file 1: Figure S1.** Anti-α-tubulin staining of a *I. australiensis* larva at 20 dpf. Video file showing a dorsal view of the anterior end. Special focus is given to the innervation of the larval dorsal hump. (MP4 2211 kb)



**Additional file 2: Figure S2.** Anti-α-tubulin staining of a *I. australiensis* larva at 26 dpf. Video file showing a dorsal view of the anterior end. Special focus is given to the innervation of the larval dorsal hump. (MP4 2222 kb)



**Additional file 3: Figure S3.** Anti-α-tubulin staining of a *I. australiensis* juvenile after metamorphosis. Video file showing a dorsal view of the anterior end. Special focus is given to the innervation of the larval dorsal hump. (MP4 2355 kb)


### Presence of the myoinhibitory peptide (MIP) in the median organ of Sabellariidae

**Fig. 4:**
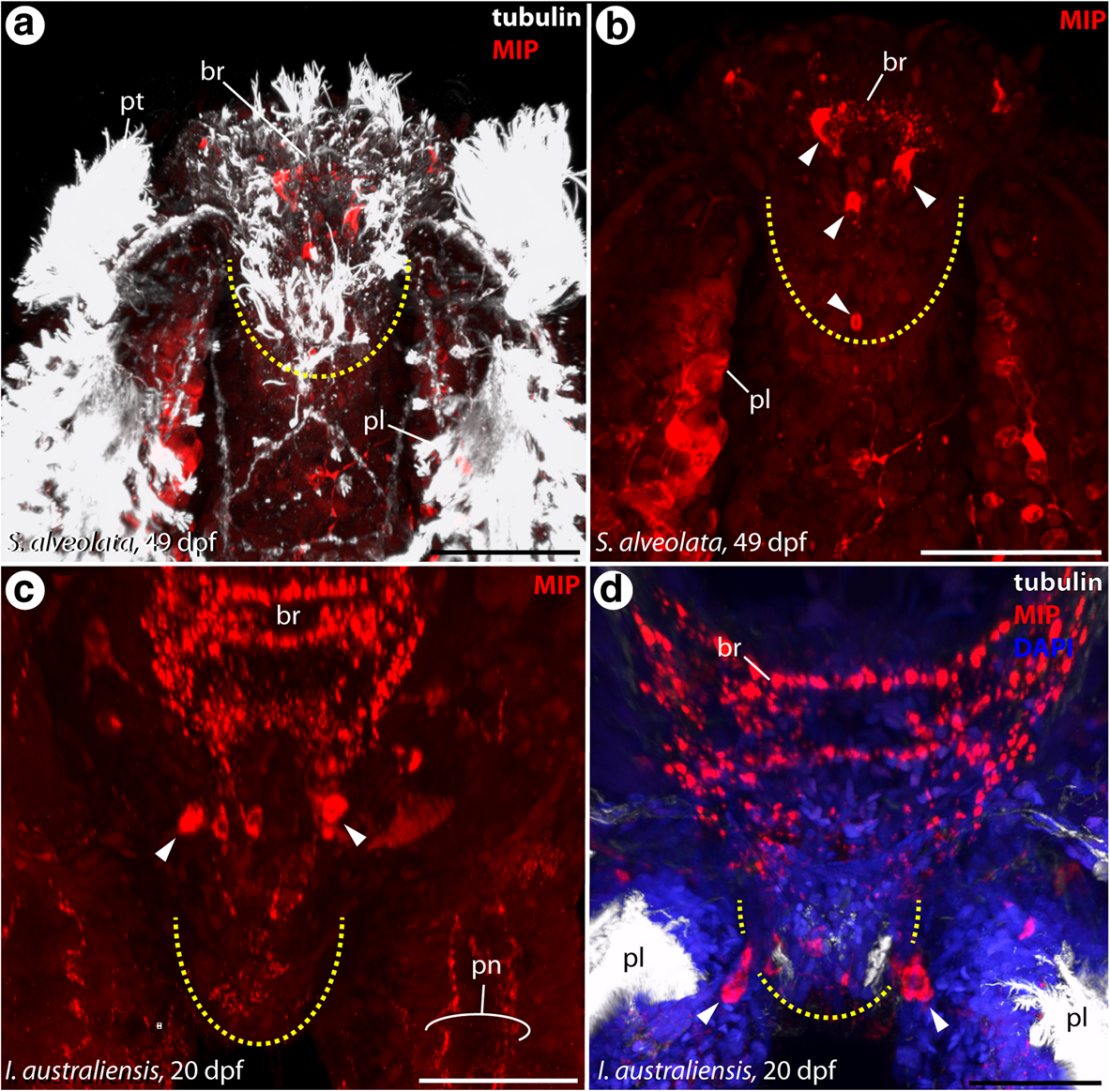
Occurrence of myoinhibitory peptide (MIP) in the median organ of Sabellariidae. Confocal maximum projections of α-tub-LIR (white), MIP-LIR (red) and DAPI (blue) staining. Anterior is up in all images. All views are from dorsal, except of (**c**) and (**d**) which are apical views. The shape of the median organ is indicated by dashed lines and prominent immunoreactive perikarya are marked with arrowheads. Age of the larvae is given in days post fertilization (dpf) and the species identity as well as the colour code for the shown staining is specified in the respective figure. **a** Distinct MIP-LIR is exhibited close to the brain (br), at the base of the median organ and along the palps (pl). **b** The median organ tip exhibits immunoreactive somata as well. **c**, **d** An anti-MIP staining without counterstaining illustrates the position of the prominent somata with MIP-LIR at the base of the median organ. br, brain; nm, neurite bundle innervating the median organ; pl, palps; pt., prototroch. Scale bars = 80 μm

The presence of the MIP was detected in median organ-bearing larval stages of both species via immunohistochemistry. Based on this investigation MIP-LIR is present in the brain and the median organ of pre-metamorphic sabellariid larvae, and along the palps (Fig. 4a-c). In particular, distinct perikarya (neuronal cell bodies) exhibiting MIP-LIR are observable at the base of the median organ (Fig. 4b-d). Further on, immunoreactive somata are present in close proximity to the ciliated tufts, the latter of which is depicted with dashed circles in the respective figure (Fig. 4c, d), at the tip of the median organ (Fig. 4b, c), as well as along the developing palps (Fig. 4b, c). Counter stainings against α-tubulin and DAPI reveal the proximity of the MIP-positive structures to the neurite bundles innervating the median organ and the presence of a nucleus as part of the respective structures (Fig. 4a, d).

### Structure of the ciliated pits and photo-sensitive structures along the median organ

**Fig. 5:**
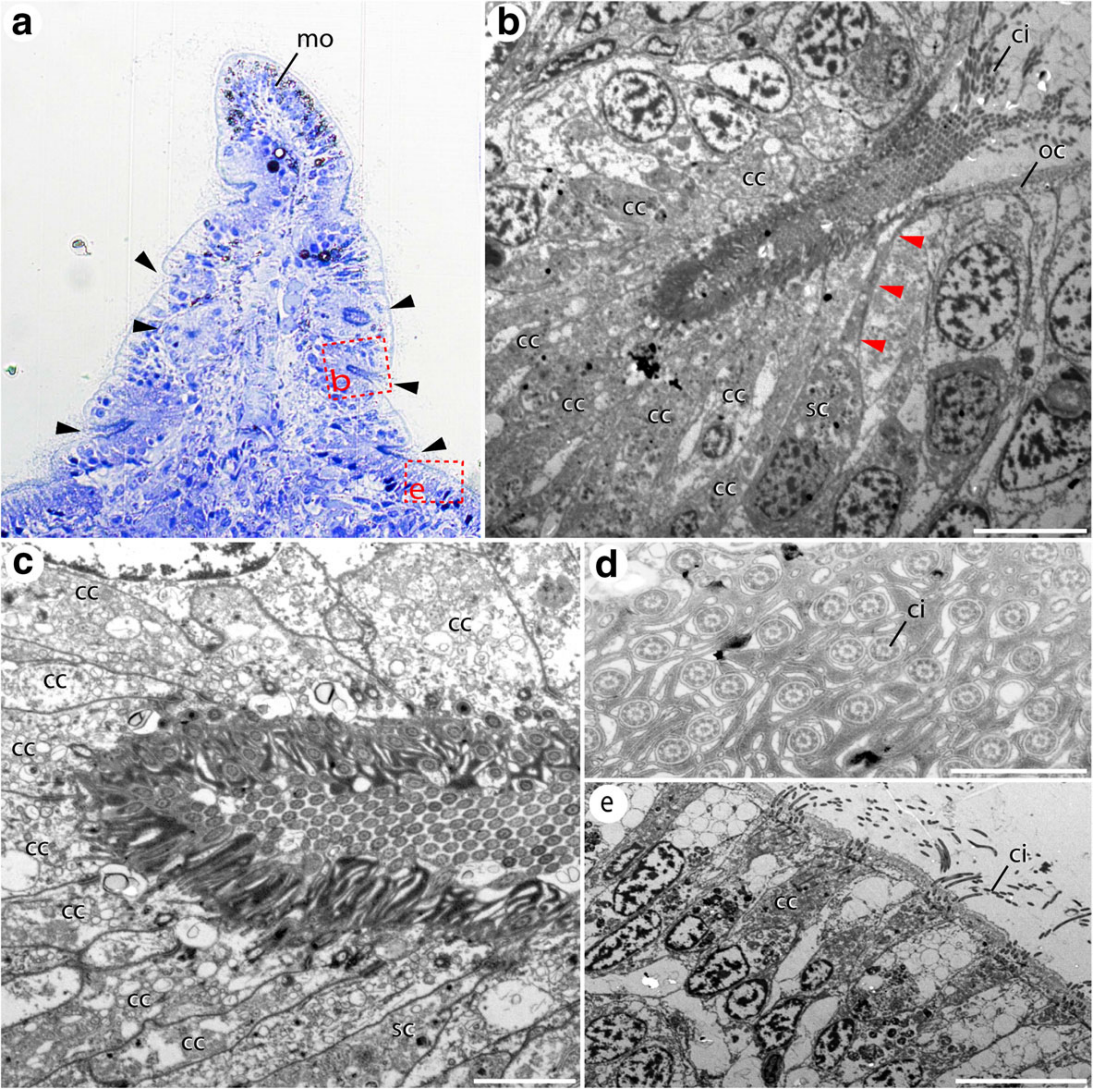
Semi-thin sections and electron microscopic images of the median organ of adult *Sabellaria alveolata*. Toluidin-blue staining (**a**) and electron microscopy (**b**-**e**). **a** An antero-posterior section of the adult median organ (mo) reveals the presence numerous ciliated pits (arrow heads) at the base of the structure. The red dashed squares indicate the position of the images shown in (**b**) and (**e**). **b** A higher magnification of a ciliated pit reveals the presence of numerous ciliated supporting cells (cc) surrounding the epidermal depression, and sending cilia (ci) out of the opening of the pit. A sensory cell (sc) situated near the pit opening sends a distinct cellular process (red arrowheads) towards the epidermal surface. The olfactory chamber (oc) is located in the region where the cellular process reaches the surface. **c** A close-up supporting cells (cc) present the density of cilia running into the epidermal depression of the pit. **d** A section showing the inner surface of the pit reveals a dense pattern of cilia (ci) and microvilli. **e** Close to the pit, but outside the epidermal depression, numerous ciliated supporting cells (cc) cover the epidermal surface. cc, ciliated supporting cell; mo, median organ; sc, sensory cell. Scale bars = 10 μm (**b**, **e**), 2 μm (**c**) and 500 nm (**d**)

**Fig. 6:**
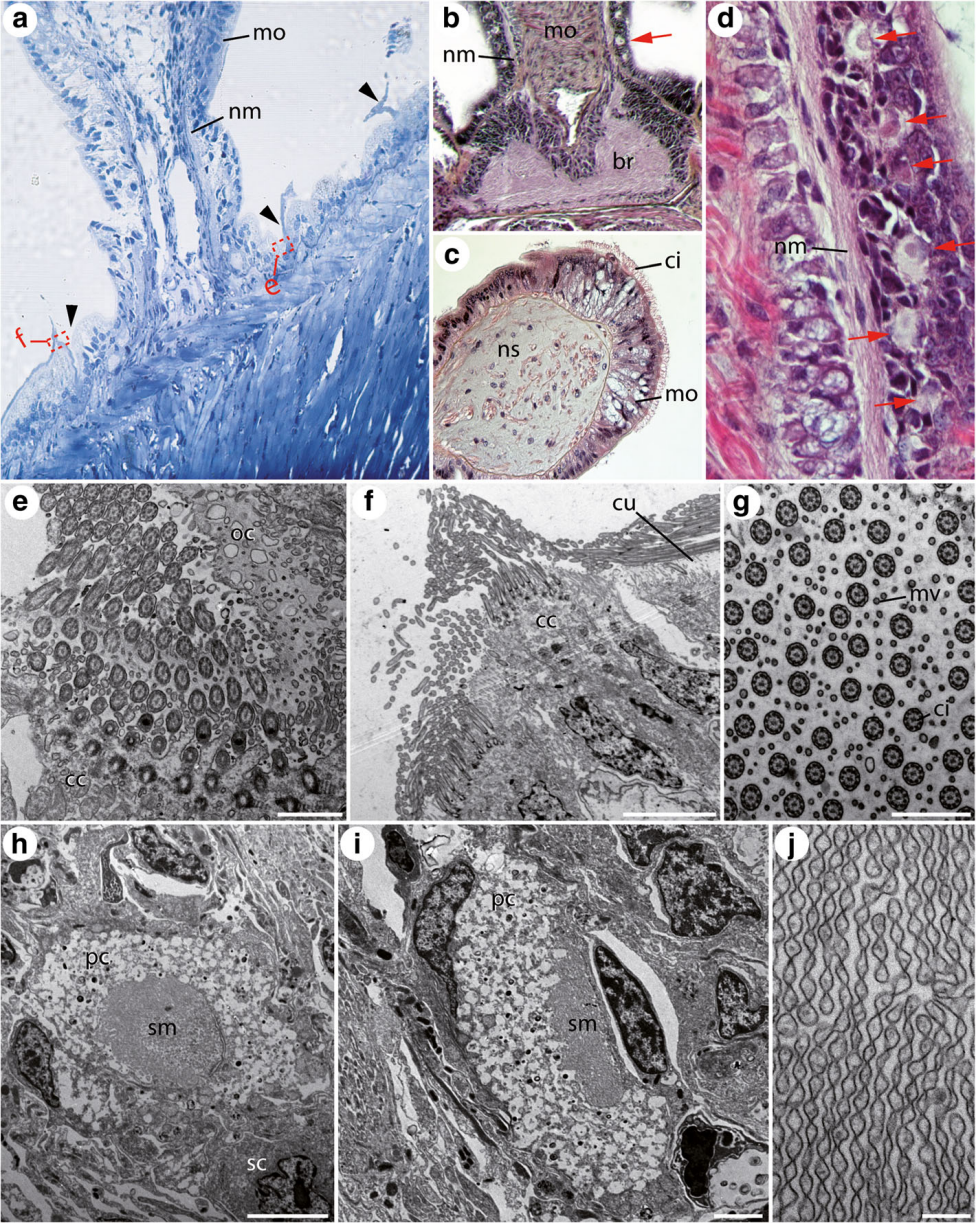
Semithin sections and electron microscopic images of the median organ of adult *Idanthyrsus australiensis*. Toluidin-blue staining (**a**), HES staining (**b**-**d**) and electron microscopy (**e**-**j**). **a** An antero-posterior section of the adult median organ (mo) reveals the presence of numerous ciliated pits bearing ciliary tufts (arrow heads) at the base of the structure. The red dashed squares indicate the position of the images shown in (**f**) and (**e**). **b** An anterior-posterior section at the base of the median organ (mo) exhibits the distinct neurite bundles (nm) innervating the (mo). The red arrow marks the position of ocelli. **c** The tip of the median organ (mo) possess a dense ciliation (ci) and a prominent nervous system innervation (ns). **d** Under higher magnification the proximity of photo-sensitive ocelli (red arrows) and the neurite bundles (nm) innervating the (mo) becomes obvious. **e** An electron microscopic image of the ciliated pit shows presence of numerous ciliated supporting cells (cc) at the base of the epidermal depression and an olfactory chamber (oc) in close proximity. **f** The area surrounding the ciliary pits is covered with cilia originating from numerous ciliated supporting cells (cc). **g** A cross section of the inner surface of the ciliated pit reveals a dense mixture of cilia (ci) and microvilli (mv) covering the surface of the supporting cells. **h**, **i** An electron microscopic image of the ocelli reveals presence of a pigment cell (pc) and a sensory cell (sc) sending the sensory membrane (sm) towards the pigment cell cup. Note that the pigment granules are not visible due to fixation artefacts. **j** The rhabdomeric-like sensory membrane can be described as microvillar, but with a whorled appearance. br, brain; cc, ciliated supporting cell; ci, cilia; cu, cuticle; mo, median organ; mv, microvilli; nm, neurite bundle innervating the median organ; ns, nervous system; oc, olfactory chamber; pc, pigment cell forming the pigment cup; sc, sensory cell; sm, sensory membrane. Scale bars = 1 μm (**e**, **g**), 5 μm (**f**, **h**), 2 μm (**i**) and 200 nm (**j**)

Regardless the different size and apparent external morphology of the median organ in both species studied, distinct similarities in the anatomy were encountered. In adult specimens of both species, numerous prominent ciliated pits are situated at the base of the median organ. In *S. alveolata*, the distinct ciliated pits are present at the base of the median ridge (= median organ), where they are arranged in parallel on both sides of the median organ base (Fig. 5a). In *I. australiensis*, several distinct ciliated pits are present next to the digit-shaped median organ (Fig. 6a). In both cases assessment concerning the exact number of pits is not possible based on the current dataset. Nevertheless, the tip of the median organ in both species bears a prominent and dense ciliated patch as well (Figs. 5a, 6c).

Ultrastructural investigations of the ciliated pits in *S. alveolata* reveal a similar composition along and close to the median organ base. Thus, each basal ciliated pit is formed by numerous multi-ciliated supporting cells, with cilia confined within a surrounding epidermal depression (Fig. 5b, c). A cross-section of the inner surface of such epidermal depression reveals a mix of cilia and microvilli covering the apical surface of the supporting cells (Fig. 5d). Furthermore, at least one sensory cell is sending a distinct receptive process towards to opening of the ciliated pit, where an olfactory chamber is situated (Fig. 5b). The entire complex of the ciliated pit is surrounded by numerous ciliated supporting cells covering the epidermal surface in close proximity to the pit (Fig. 5e).

In *I. australiensis* the situation is comparable. The ciliated pits are also formed by multi-ciliated supporting cells sending their cilia into an epidermal depression with an olfactory chamber located close by (Fig. 6e). Additional supporting cells are surrounding the entire complex (Fig. 6f). A view of a cross-section of the inner surface of such a depression reveals a dense pattern of microvilli and cilia covering the surface of the supporting cells (Fig. 6g). Unfortunately, the position of the sensory cell(s) could not be revealed based on the current data.

Adults of *I. australiensis* have another remarkable sensory complex being part of the median organ complex. Prominent ocelli are located along the sides and base of the median organ (Fig. 6b, d). These ocelli are composed of two cells; a pigment cell forming the pigment cup and a sensory cell projecting a rhabdomeric-like sensory membrane into the centre of the pigment cup (Fig. 6hi, i). There are no obvious lenses or other optical structures. Pigment granules are apparently lost in fixation, resulting in a transparent pigment cup in micrographs (Fig. 6h, i). Interestingly, the putative photoreceptive membrane is unlike any other example known as described in the literature so far [36]. It appears to be microvillar in nature, but the whorled microvilli are joined by narrow cytoplasmic channels running their length (like webbing between sheets of corrugated membrane stacked together) (Fig. 6j).

## Discussion

The sabellariid median organ is a structure frequently referred to in taxonomic descriptions but is a scarcely investigated character complex [18] with putative sensory function and involvement in chemosensation [8].

The investigated sabellariid adults bear numerous prominent ciliated pits located along the median organ. Ultrastructural investigations of the latter ciliated pits revealed distinct similarities with nuchal organs of other polychaetes. Thus, ciliated supporting cells forming a ciliated tuft embedded in an epidermal depression together with closely related sensory cells and an olfactory chamber are characteristics of annelid nuchal organs [23, 37] (see also Fig. 1 for comparison). Notably, the herein presented data do not allow a detailed comparison of the sensory cells themselves and their neuronal connection. Nevertheless, a comparable organization of the nuchal organs and a location inside an epidermal depression is documented in the putative sabellariid sister group, the Spionidae [38]. For example, in *Spio* cf. *filicornis* (Müller, 1776), the nuchal organs consist of numerous ciliated supporting cells forming the ciliary tuft, a sensory cell sending a prominent process towards the epithelial surface close-by and an olfactory chamber hosting the receptive apical part of the sensory cell [25]. Notably, the investigated Spionidae exhibit nuchal organs along their caruncle region. Several spionid taxa even seem to exhibit more than one pair of nuchal organ-like structures [24]. The same is true for the ciliated pits in sabellariids, which are present at the base of the median organ. The presence of similar ultrastructural details in the sabellariid ciliated pits provides a strong support for a homology of the ciliated pits in Sabellariidae, the nuchal organs in Spionidae and the remaining Annelida. Nonetheless, it has to be stated that annelid nuchal organs usually bear numerous sensory cells whereas our investigations solely exhibit the presence of one or at least few – a fact that can be also owed to the limited amount of ultrastructural data for the herein investigated Sabellariidae. In line with previous descriptions, our analyses of larval stages furthermore support a developmental origin of the sabellariid ciliated pits in the region of the dorsal prototroch gap. Thus, late larval stages of both investigated species bear prominent ciliary tufts at the base of their dorsal hump, which can be traced throughout metamorphosis and are still present in juvenile and adult specimens. The same is reported for the spionid *Pygospio elegans* Claparède, 1863 [39]. Accordingly, the spionid nuchal organs develop as ciliated tufts in the dorsal anterior region of late larvae, and the ultrastructure of the larval and adult structures can be assumed as being comparable and without prominent changes based on earlier analyses [20, 26, 39–41]. Furthermore, a change of the life mode does not seem to be reflected in drastic (ultra-) structural changes of the nuchal organ organization [41]. In *Pygospio* prominent ciliated tufts located posterior of the dorsal gap of the larval prototroch later become part of the adult caruncle and exhibit the adult nuchal organ. Based on our results, the sabellariid larval ciliary tufts located posterior to the dorsal gap of the prototroch become incorporated into the adult median organ and seem to develop into the adult nuchal organ-like ciliated pits. Besides a comparable ultrastructure and development, another striking similarity between the nuchal organ-like structures in sabellariids, and the nuchal organs in spionids and the remaining Annelida, their respective neuronal innervation is highly comparable. Nuchal organs are innervated from neurite bundles branching off from the dorsal roots of the circumesophageal connectives within the annelid brain [19, 20, 42]. For *Pygospio* a distinct innervation of the larval and adult nuchal organs is present via neurite bundles originating from the dorsolateral brain [39, 40]. So far not described for Sabellariidae [29], our data reveal the presence of such innervating neurite bundles running from the dorsolateral brain (in close proximity to the dorsal root of the circumesophageal connective) towards the dorsal ciliated tufts in late larval stages. Examination of the same – but already modified - region in juvenile worms after metamorphosis shows a similar innervation pattern of the ciliated tufts within the median organ. Thus, the larval ciliary tufts and the ciliated pits of adults can be assumed as being homologous structures. A putative existence of the sabellariid nuchal organs at the base of the adult palps [29, 43] can be rejected due to our investigations. The possible homology of the ciliary structures along the median organ of members of Sabellariidae and the nuchal organs of other polychaetes had recently been anticipated by Faroni-Perez et al. [18] who suggested that the ciliation observed in larval median organ may function to promote and detect seawater circulation and to mediate faster chemical signal delivery to the sensory structures, including the sensory cilia, but ultrastructural support for such a hypothesis was lacking.

Although presumed to be involved in chemosensory functions or reproductive purposes, the role of nuchal organs was always deduced only from ultrastructural data [20, 24] and evidence for such hypotheses was lacking so far. Recently, physiological investigations in the nereid *Platynereis dumerilii* verified such a chemosensory role for the annelid nuchal organ [28]. For the first time, our investigations reveal the presence of the myoinhibitory protein MIP in the region of the larval median organ and in close proximity to the larval nuchal organs in late larval stages of both examined species. Known to be involved in the regulation of insect ecdysone and juvenile hormone levels [44–46], in settlement behaviour in cnidarians [47] and in the activation of ingestion and gut peristalsis in Annelida [48], MIP is also well-known for its involvement in larval settlement behaviour in *P. dumerilii* (Audouin & Milne Edwards, 1833) [49]. Hence, the neuropeptide MIP (or allatostatin-B) is also expressed in chemosensory-neurosecretory perikarya closely related to the apical organ in larval *Platynereis*. Together with a *Drosophila*-like sex ligand receptor, MIP causes rapid settlement of competent larvae [50].

In the case of the herein investigated sabellariids *S. alveolata* and *I. australiensis*, MIP-LIR was detectable throughout the larval brain, within distinct cells along the developing palps, but also in few prominent perikarya located inside the larval median organ. The presence of such putatively chemosensory-neurosecretory MIP-positive perikarya within the median organ and in close relation to the nuchal organ-like ciliary structures hints towards a possible role of the entire median organ complex, including the ciliated pits, in sensing of chemical stimuli and provides further support for an involvement of the latter structure in larval settlement – a hypothesis which was proposed earlier [18], but which still requires further investigation. Since MIP is known to have various functions [44–48], a final statement concerning its role during sabellariid development can only be made after functional analyses. Notably, the presence of MIP-LIR in the larval brain and along the palps cannot be explained based on our current data.

Besides the putative nuchal organs, data presented herein reveal the presence of an unusual type of photoreceptor as another part of the median organ in *I. australiensis* adults. In general, the ocelli resemble the simple pigment cups that form the cerebral ocelli of other polychaetes [23]. The corrugated sensory membrane ultrastructure, however, is unlike those observed in the segmental ocelli of sabellids [50] or *Platynereis* [51], the pygidial [52], or the cerebral receptors of sabellids [52], serpulids [53], and other polychaetes. Further investigations are necessary to understand the reason for such an enigmatic structure of the sensory membrane.

Presumably, these ocelli function similarly to the eyes and ocelli found on the anterior, prostomial-derived radioles of adult sabellids and serpulids, which detect the shadows of encroaching threats and initiate a startle response [54]. However, the radiolar eyes are composed of ciliary photoreceptors that express c-type opsin photopigments in sabellids and serpulids [53, 55–57], suggesting that these microvillar-like sabellariid photoreceptors are a convergent emergence of unique derivation, perhaps influenced by disparate ontogeny of the apical sensory structures between these groups.

## Conclusion

For the first time, the herein presented data reveal strong evidence concerning a nuchal organ affinity of the sensory ciliated pits located at the sabellariid median organ and provide further insights into the evolution of annelid sensory organs. Furthermore, our investigations illuminate the so far scarcely examined development of nuchal organ-like sensory structures in Annelida and refute a presence of nuchal organs at the base of the palps in Sabellariidae. Instead, external morphology, neuronal innervation, developmental fate and ultrastructural details of the discovered median organ-based ciliary structures are comparable with the characteristics known for nuchal organs in Annelida and therefore indicate a possible homology of the sabellariid ciliated pits and nuchal organs of other polychaetes. Notably, the presence of myoinhibitory peptide (MIP) in the median organ and in close relation to the ciliated pits in late larvae points to the possibility of an involvement of the entire sabellariid median organ complex including the prominent ciliated pits in particular into chemo-sensation and larval settlement behaviour. To clarify whether the observed nuchal organ-like ciliated pits can be called nuchal organs and are involved in larval settlement, additional investigations including further ultrastructural investigations and functional experiments are necessary.

## Abbreviations

α-tub: Acetylated-α-tubulin
dpf: Days post fertilization
MIP: Myoinhibitory peptide/allatostatin-B
LIR: Like immunoreactivity
